# Analysis of Agile Canine Gait Characteristics Using Accelerometry

**DOI:** 10.3390/s19204379

**Published:** 2019-10-10

**Authors:** Hasti Hayati, Fatemeh Mahdavi, David Eager

**Affiliations:** School of Mechanical and Mechatronic Engineering, Faculty of Engineering and IT, University of Technology Sydney, Ultimo, NSW 2007, Australia; sanaz.mahdavi@uts.edu.au (F.M.); david.eager@uts.edu.au (D.E.)

**Keywords:** accelerometry, wavelet transform, fast fourier transform, canine, gait, greyhounds

## Abstract

The high rate of severe injuries associated with racing greyhounds poses a significant problem for both animal welfare and the racing industry. Using accelerometry to develop a better understanding of the complex gait of these agile canines may help to eliminate injury contributing factors. This study used a single Inertial Measurement Unit (IMU) equipped with a tri-axial accelerometer to characterise the galloping of thirty-one greyhounds on five different race tracks. The dorsal-ventral and anterior-posterior accelerations were analysed in both the time and frequency domains. The fast Fourier transform (FFT) and Morlet wavelet transform were applied to signals. The time-domain signals were synced with the corresponding high frame rate videos of the race. It was observed that the acceleration peaks in the dorsal-ventral accelerations correspond to the hind-leg strikes which were noted to be fifteen times the greyhound’s weight. The FFT analysis showed that the stride frequencies in all tracks were around 3.5 Hz. The Morlet wavelet analysis also showed a reduction in both the frequency and magnitude of signals, which suggests a speed reduction throughout the race. Also, by detecting abrupt changes along the track, the wavelet analysis highlighted potentially hazardous locations on the track. In conclusion, the methods applied in this research contribute to animal safety and welfare by eliminating the factors leading to injuries through optimising the track design and surface type.

## 1. Introduction

Sensors have been widely used in the field of biomechanics for different purposes and not only limited to clinical practices [1,2] and gait characteristics [1,3,4,5,6]. Inertial Measurement Units (IMU), which are usually equipped with accelerometers, gyroscopes and magnetometers [7], can be used to study complex dynamics such as turning [8,9], locomotion on difficult terrains [10] and amusement rides [11] where conventional methods such as force-platforms cannot be deployed.

For instance, Wilson et al. studied the hunting dynamics of cheetahs using an in-house IMU in the shape of a collar. They successfully analysed the locomotory dynamics of cheetahs hunting in the wild [12]. IMUs can also be used to study the locomotion kinetics of arboreal animals that cannot be studied through force platforms. In a study conducted by Bayren et al. a tri-axial accelerometer was used to measure the landing and gliding kinetics of the Malayan colugo. They found that the propulsive kinetics during take-off increase in longer glides. However, the landing forces decrease with longer glide-distances [13]. Spence et al. used a custom designed accelerometer backpack to study the effect of complex terrain (leaf litter) on the locomotion dynamics of rapid insects (Death’s head cockroaches). They attached the accelerometer backpack close to the insect center of mass (CoM). Their results showed that the peaks of CoM dorsal-ventral acceleration on soft surfaces were smaller than on rigid surfaces [10].

Use of accelerometers to characterise equine gaits, to the best of our knowledge, dates back 25 years to studies conducted by Barry et al. where they used a single IMU to analyse equine gait [1,14]. Later on, in a study conducted by Uchiyama et al. in 2011, the walking acceleration patterns in 50 healthy human subjects were compared with those of 11 horses to examine whether the movement of the horse’s pelvis during horseback riding resembles human walking. The tri-axial acceleration data (dorsal-ventral, medial-lateral and anterior-posterior) were analysed by FFT. Witte et al. used two uni-axial IMUs, one mounted on the hoof and another on the metacarpophalangeal joint and measured the footfall timing of horses (Equus caballus) to predict the peak ground reaction forces (GRF) acting on limbs. They compared their results with force plate data which showed a mean error of 2.3 ms and 3.5 ms for the timing of limb touch-down and lift-off, respectively, across all gaits [3]. Following this study, Pfau et al. used three different sensors to measure the galloping dynamics of thoroughbred racehorses, in particular, their CoM movement and external energy fluctuation [15]. One accelerometer was mounted on the horse’s hoof to measure the contact time of the limb with the ground. A global positioning system (GPS) data logger and an IMU sensor, which were needed to estimate the CoM movement as well as estimating the external mechanical energy, were mounted on the wither of the animals. Their results showed that mechanical energy is mostly affected by changes in craniocaudal velocity (anterior-posterior direction). It is found that the CoM movement was also negligible during a high-speed gallop.

In the a study conducted by Jenkins et al. a single wearable inertial sensor, mounted above the carpal joint on the lateral side of the fore-limb, was used to determine and then automate the temporal gait characteristics in canines. Their results were manually validated with the high-frame-rate (HFR) videos of the canines and showed acceptable accuracy [16]. In another study conducted by Rhodin et al. [17] an inertial sensor-based system is used in canines to detect and quantify induced moderate lameness as well as differences between supporting and swinging limb lameness. The Alvarez et al. study proved that canine lameness can be detected by measuring the vertical head and pelvic movement [18]. However, motion-capturing methods are time-consuming and are mostly limited to the laboratory environment. The results of the Rhodin et al. study showed that by using three inertial based sensors attached to the mid-line of the top of the head, the mid line of the spinous processes of the second sacral vertebra and the dorsal surface of the metacarpal bones of the forelimb, the moderate induced lameness in canines can be detected and quantified [17]. In a similar study conducted by Ladha et al. [19], an IMU equipped with a tri-axial accelerometer and gyroscope coupled with a standardised walking course, was used to detect canine lameness. The unit was then attached to each leg of the dog for data collection. The results showed that the unit was capable of precisely delineating the step time as well as the toe-touch and toe-lift. Gerencsér et al. also used a single inertial sensor to characterise the locomotion behavior in freely moving dogs [20]. Britt et al. also used a commercial IMU (Blackthorn K9 Equipment Inc., Monroe, MI, USA) embedded on a vest equipped with GPS sensor, magnetometer, gyroscope and accelerometer for real-time position and orientation tracking of canines [21].

Greyhounds are a breed of canine belonging to the family of sight hounds [22]. They have been used in racing activities throughout history because of their unique sprinting capability. Due to the inherent hazards of racing [23] as well as environmental factors such as track designs [24,25,26], the rate of injuries, specifically muscloskeletal injuries of the rear legs, in these canines is high [23,25,26,27,28]. For instance, in the latest report published by the Greyhound Welfare and Integrity Commission (GWIC) New South Wales in the second quarter of 2019 the total injury rate was 32.9 per 1000 greyhounds raced. In another report published by GWIC a total of 2034 greyhounds were reported injured from July 2018 to March 2019 [29].

Thus, a better understanding of the greyhound racing gait as well as analysing the interaction of greyhound limbs and the track surface, will assist in understanding the injury mechanisms. As mentioned above, IMU technology is one of the best tools to study animals gait in-situ. The results obtained from accelerometry would assist in spotting dangerous locations on race tracks, which can be caused by track design and surface properties and eventually eliminating them from the race to improve the safety of a race.

In this work, in order to characterise the galloping gait of racing greyhounds using accelerometry, a total of thirty-one greyhounds ran on five different racing tracks. The in-house IMU was mounted approximately on top of the animal CoM in a racing jacket with a pocket to accommodate the sensor. Two experiments were designed in this work. In the first experiment, the time-domain accelerations in both anterior-posterior and dorsal-ventral directions were synchronised with the HFR videos of the same run to match the corresponding gait events with accelerations. The second experiment aimed to study the effect of turning and surface compliance on greyhound galloping dynamics. It was found that utilising a single IMU was beneficial in characterising the temporal gait characteristics of greyhound galloping gait, namely the stride and step frequency. These parameters were obtained by deploying FFT and wavelet transforms on acceleration data, which is explained in detail in the following sections.

## 2. Materials and Methods

### 2.1. Race Tracks Specifications

Five greyhound race tracks were included in this study with the specifications tabulated in the following Table 1:

### 2.2. IMU Specification

The first IMU was a commercial IMU (GPSports/SPI Pro X) capable of measuring accelerations up to 16 G at a sampling frequency of 100 Hz. The second IMU was an in-house IMU package, called Integrated kinematic measurement system (iKMS), equipped with a 185 Hz tri-axis accelerometer capable of measuring acceleration up to 16 G. The iKMS allows tracking of tri-axial body rotation, tri-axial linear body acceleration and tri-axial magnetic heading. For this paper, only the tri-axial linear accelerations were analysed.

### 2.3. Experiment 1: Identification of the Corresponding Limb Acceleration

**Figure 1:**
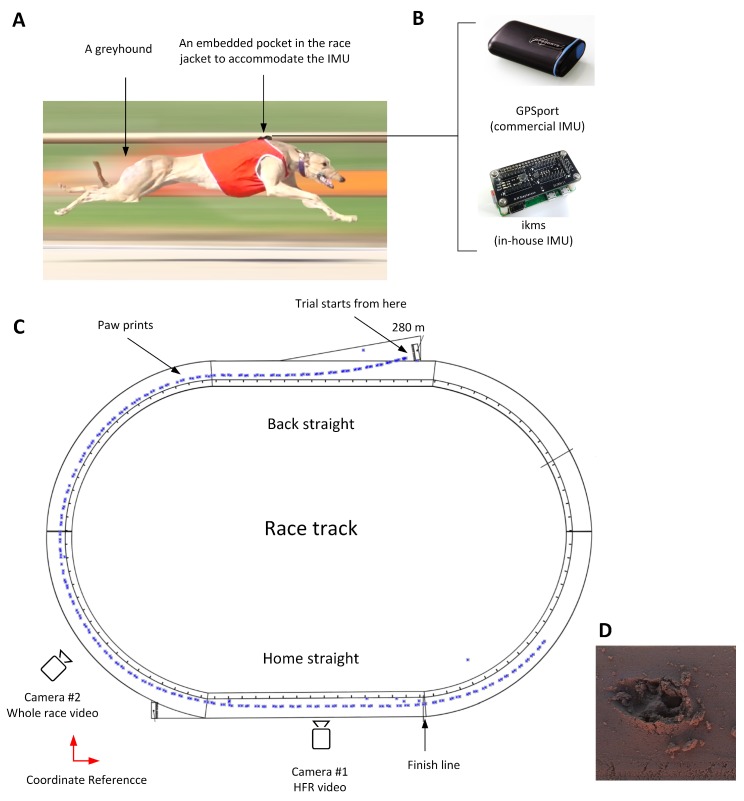
A greyhound galloping on the straight section of a track with sand surface and wearing the modified jacket with Inertial Measurement Unit (IMU) pocket (**A**). The commercial (GPSports/SPI Pro X) and in-house IMU (shown here without its protective cover) (**B**). The schematic view of the oval-shaped sand track and the greyhound’s paw prints marked in blue. The coordinate reference depicts the location of the theodolite that was used to survey the paw prints. Two cameras are mounted for simultaneous kinematic video capture (**C**). Greyhound’s paw print (**D**).

The first experiment was designed to synchronise greyhound galloping gait events with the acceleration signals measured by the IMU. In other words, this experiment aimed to match the IMU signals, that is, linear acceleration in the anterior-posterior and dorsal-ventral direction with each limb strike. The commercial IMU (GPSports/SPI Pro X) with the above mentioned specifications was used for this experiment. A greyhound was encouraged to run in a training session on Track E (Figure 1A) over a sandy surface. The sensor was placed into a sewn pocket located on top of the greyhound’s wither which is located approximately above the CoM [30]. The schematic of the experiment is depicted in Figure 1C.

The trial started from 280 m boxes, followed by the Back Straight to a turn of 52 m radius and then the Home straight. Owing to ethical considerations, only a single dog raced in the trial session. The paw print surveying started immediately after the greyhound was collected by the owner. A theodolite was located outside the track (the coordinate reference is shown in Figure 1C).

A simultaneous kinematics study was done with two Sony DSC-RX10-III cameras, one set to 50 fps to capture the whole race video, the other set to 500 fps to capture at least two full strides of the greyhound with greater resolution. The HFR camera was mounted close to the finish line to allow greyhounds to achieve their highest speed and steady-state gallop.

### 2.4. Experiment 2: Effect of Turning and Surface Compliance on the Galloping Gait Characteristics

In order to examine whether different sections of the track and surface type are distinguishable in the acceleration data, the acceleration peaks (both negative and positive) of greyhounds galloping on two tracks of similar shape (oval-shaped) but different surface types, were compared. In other words, this experiment aimed to examine whether (i) different sections of the race track (bend and straight sections) affect greyhound galloping dynamics and (ii) whether galloping over different types of surfaces (sand and grass surface) has an impact on greyhound galloping gait. Tracks A and C were selected for conducting these experiments as they were both oval shaped and had different surface types and more importantly, the kinematic data (video of the whole race) were available on both tracks. Accordingly, owing to the recorded videos of trials, running on straight and bend sections could be spotted on the acceleration signals recorded by the IMU.

Greyhounds were chosen randomly to run in this experiment. Local trainers provided those greyhounds which were familiar with the respective tracks.

Four individual greyhounds ran on Track A. Track A was an oval-shaped track with a large radius of 81 m and a grass surface. The bends were essentially level which meant the camber angle was almost 0${}^{\circ }$. The track had two race distances, 300 m and 400 m. Two greyhounds ran from the 300 m distance (starting on a bend, followed by a Home straight and finish line.) and the rest ran from the 400 m distance (starting on Back straight, followed by bend, Home straight and finish line).

Four greyhounds ran individually on Track C. Track C was an oval-shaped sandy track, with a turn radius of 53 m. The track had four race distances, 305 m, 407 m, 480 m and 610 m. Two greyhounds ran from the 407 m distance (starting in Back straight, followed by a bend, Home straight and finish line.) and the rest started from the 480 m distance (starting in bend, followed by Back straight, second bend, Home straight and finish line).

In both Track A and C, an in-house iKMS sensor with a 185 Hz tri-axis accelerometer was used. To meet the first aim of this experiment (effect of bend and straight section on greyhound galloping dynamics), we compared the anterior-posterior and dorsal-ventral accelerations of six consecutive strides on the bend (approximately bend apex) with those of the Home straight, of each greyhound.

In order to see the effect of surface compliance on the dynamics of galloping greyhounds, which was the second aim of this experiment, anterior-posterior and dorsal-ventral acceleration of six consecutive strides of galloping in the Home straight of Track A, were compared with those of Track C.

#### 2.4.1. The Temporal Galloping Gait Characteristics Using FFT

Stride frequency and stride length are two important speed indicators in legged locomotion. The higher the stride frequency and the stride length, the higher the speed [31]. Stride frequency can be measured through optical-based systems (by measuring the footfall timing via videos). However, analysing optical-based data is time-consuming, compute-intensive and limited to a laboratory environment [17]. Alternatively, accelerometry can be used to measure the stride frequency by applying FFT spectral analysis on acceleration signals.

FFT is a powerful tool in signal processing, which is applied in numerous fields [32]. The FFT is based on the discrete Fourier transforms (DFT) but is much faster than DFT. The DFT is defined as:

(1) $$X\left(k\right)=\sum_{n=0}^{N-1}x\left(n\right)e^{-j2\pi n/k},k=0,1,\ldots ,N$$

where x(n) is a finite data sequence in the time-domain and consists of *N* elements and $e^{-j2\pi n/k}$ is a primitive *N*th root of 1 [33].

All gaits at a steady-state condition (i.e., constant speed) can be considered as a sum of their stationary periodic motion. Deploying FFT spectral analysis would assist in determining the main harmonics of the locomotion which contains information regarding the fundamental temporal gait characteristics that is, the stride and step frequency. Based on Barry et al. study, which aimed to characterise equine gaits via acccelerometry, the first harmonic on the FFT of dorsal-ventral accelerations is the stride frequency of the animal [14]. Accordingly, to compare the stride frequency the FFT was applied on the dorsal-ventral accelerations for all race tracks.

#### 2.4.2. Wavelet Analysis

The FFT provides valuable information regarding the temporal gait characteristics but it is localised only in frequency. This led to the application of wavelet analysis which is localised in both frequency and time. Wavelet analysis is another strong tool in signal processing which decomposes a time-series into time-frequency space. In other words, with wavelet analysis one is able to determine both the dominant modes of variability and how those modes vary in time. The continuous wavelet transform (CWT) is a type of wavelet analysis that provides an over-complete representation of a signal by letting the translation and scale parameter of the wavelets vary continuously and is defined as:

(2) $$W_{n}\left(s\right)=\sum_{n^{\prime }=0}^{N-1}x\left(n^{\prime }\right)\sqrt{\frac{\delta t}{S}}\Psi_{0}^{*}\left[\frac{(n-n^{\prime }\delta t}{S}\right]$$

where *S* is the wavelet scale and Ψ is the wavelet translation with ∗ symbolise a complex conjunction [34].

Thus, wavelet analysis can be used for gait analysis in biomechanics [1]. Using the wavelet analysis, one can detect abrupt changes in signals, which depending on the purpose of the study, contain valuable information. For instance, in a work conducted by Biau et al. a wavelet analysis was performed on dorsal-ventral accelerations in nineteen dressage horses to determine the gait transitions [35]. Moreover, the wavelet spectral analysis was previously used to characterise jumping techniques in equines [1].

Accordingly, the wavelet spectral analysis was performed on anterior-posterior and dorsal-ventral accelerations for all race tracks to determine abrupt changes in the signals during the run which may indicate hazardous locations on the race tracks.

### 2.5. Animal Ethics Approval

Animal ethics approval was obtained (UTS ACEC ETH16-0367). No adverse effects on animal behaviour due to the wearing of the jacket were observed. It should be noted that the wearing of jackets by greyhounds is routine while they are training or racing. The weight of both IMU devices are around 70 g, making it three orders of magnitude smaller than the greyhound’s body mass of 40 kg.

## 3. Other Matters

A total of twenty-two greyhounds ran on Track B and Track D, circular and oval-shaped tracks respectively. The signals results were analysed in the following sections.

## 4. Results

### 4.1. Experiment 1: Limb Strikes and Their Corresponding Acceleration Signals

**Figure 2:**
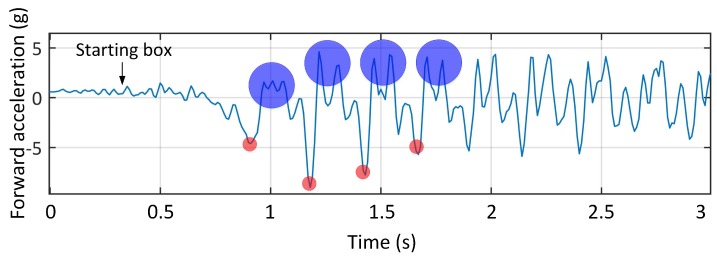
Anterior-posterior acceleration vs. time of eight consecutive strides of a greyhound. The negative peaks (highlighted by red marks) correspond to fore-leg strikes and the positive peaks (highlighted with blue marks) correspond to hind-leg strikes.

The results of the first experiment are shown in Figure 2. Using the whole race video and paw prints allowed matching the corresponding acceleration data with the leg strikes. In Figure 2, the anterior-posterior acceleration versus time for the first three seconds of the test is shown. The first negative peaks correspond to fore-leg impacts with the artificial grass which was used in front of the starting boxes to provide enough traction for animals to accelerate. The consecutive positive peaks correspond to the hind-leg impact, which provides the propulsion for locomotion [36]. It should be noted that the first two spikes in the signals do not correspond to paw print data as they were on synthetic grass. We compared our results with the Wilson et al. study which used an in-house tracking collar including a GPS and an IMU to capture the locomotor dynamics and outcome of more than three hundred predominantly hunting runs of five wild cheetahs in Botswana [12]. Their results showed that the forward acceleration peaks were approximately due to hind-leg foot contact, which is consistent with our results.

**Figure 3:**
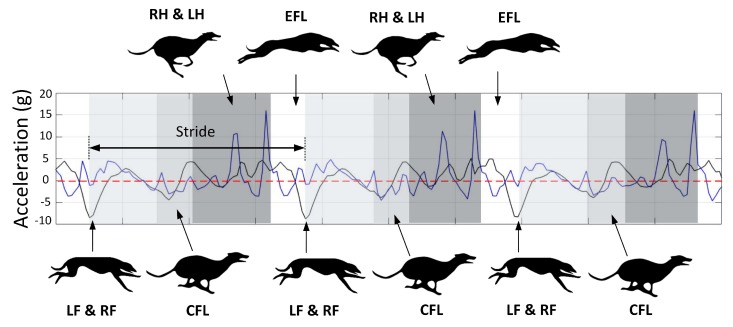
Forward (Black trace) and vertical (blue trace) acceleration vs. time of three consecutive strides of a greyhound and the corresponding gait events.

Using the HFR data, the footfall timing of each of the limbs during the rotary gallop was measured. Rotary gallop is a four-beat gait with two flight phases [37]. The pattern of limb impacts in this gait is rotating that is, left fore-leg (LF), right fore-leg (RF), compressed flight phase (CFL), right hind-leg (RH), left hind-leg (LH) and extended flight phase (EFL). The footfall timing was used to match the acceleration signals with the relevant “gait events” of the rotary gallop. The time difference between two consecutive negative peaks was one stride (approximately 300 ms). As mentioned, the negative peaks were due to fore-leg strikes. Adding the LF and RF stance duration leads to the fore-leg lift-off or beginning of the CFL. The next event is the hind-leg strike. Adding the RH and LH stance duration leads to the hind-leg lift-off or the beginning of the EFL. Finally there is another negative peak in forward acceleration which is the beginning of the next stride. We continued to use this method for the remaining signals and obtained the same pattern as evidenced in Figure 3.

### 4.2. Experiment 2: Effect of Bend and Different Surface Composition on Greyhound Galloping Characteristics

**Figure 4:**
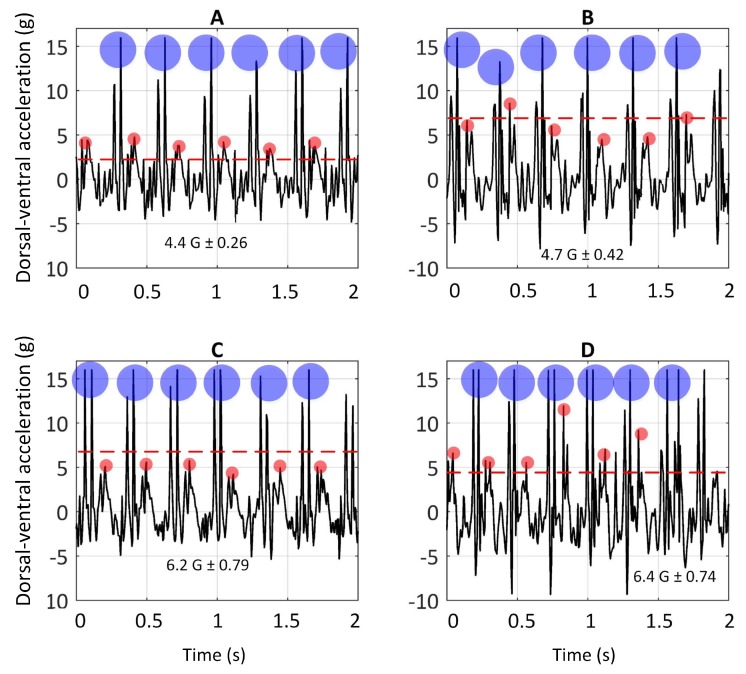
Dorsal-ventral acceleration of six consecutive strides on Home straight of Track A (**A**). Dorsal-ventral acceleration of six consecutive strides on the bend of Track A (**B**). Dorsal-ventral acceleration of six consecutive strides on Home straight of Track C (**C**). Dorsal-ventral acceleration of six consecutive strides on the bend of Track C (**D**). The blue and red circles show the hind-legs and fore-legs strikes, respectively. The red dashed lines show the average of peaks of signals due to fore-leg strikes.

Figure 4 illustrates the dorsal-ventral accelerations ( a sample of one greyhound) on the bend apex (the bend before the Home straight) and the Home straight on Tracks A and C. The rationale for picking these two sections was allowing greyhounds to have enough time to achieve a steady-state gallop. The *t*-test (paired two samples for means) was conducted. The values of *p* ≤ 0.05 were considered statistically significant. The peaks of signals (anterior-posterior and dorsal-ventral accelerations) in six consecutive strides on the bend apex with that of the Home straight, in both Tracks A and C, for four greyhounds, are compared and the results are reported in the following sections.

The dorsal-ventral acceleration of greyhounds galloping on Track A and C are given in this section. As argued earlier, the high positive peaks on dorsal-ventral accelerations were due to hind-leg strikes and the lower consecutive peaks were due to foreleg strikes, depicted in Figure 3. Comparison of accelerations upon hind-leg impacts on the bend and the Home straight showed a similar value of 15 G (1 G due to the greyhound’s weight was deducted from 16 G). In other words, forces almost fifteen times a greyhound’s body weight were acting on the limbs in the dorsal-ventral direction each time the hind-legs came into contact with the ground. This explains the high rate of hind-leg injuries in racing greyhounds [8,38]. It is worth noting that the IMU full-scale range was 16 G. No clipping of the signal was noted. Nevertheless, it is possible that there may have been higher acceleration values acting on the greyhound’s limbs.

Furthermore, significantly higher values of dorsal-ventral accelerations were seen to be acting on fore-legs when turning than when running along the Home straight on both Tracks A [t(4) = 5.7 *p* = 0.005] and C [t(4) = 8.14 *p* = 0.002].

**Figure 5:**
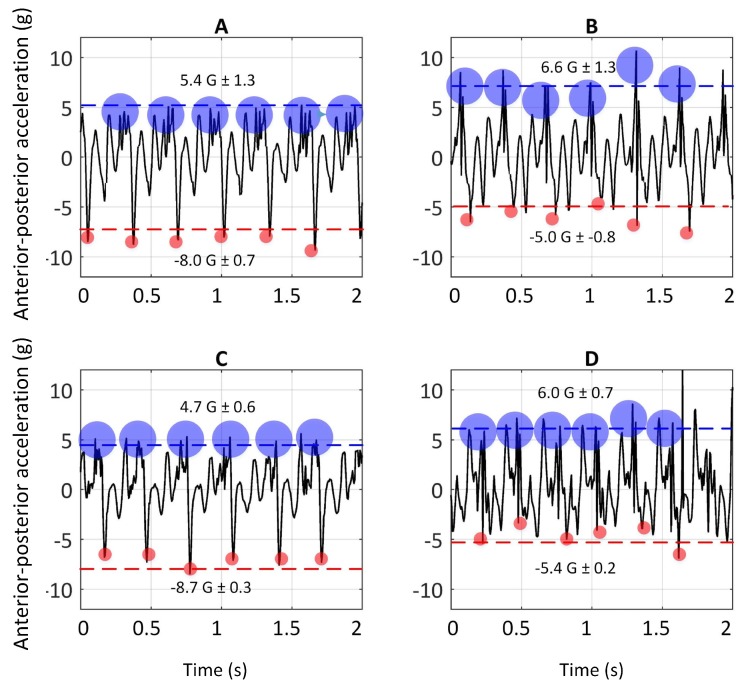
Anterior-posterior acceleration of six consecutive strides on the Home straight of Track A (**A**). Anterior-posterior acceleration of six consecutive strides on the bend of Track A (**B**). Anterior-posterior acceleration of six consecutive strides on the Home straight of Track C (**C**). Anterior-posterior acceleration of six consecutive strides on the bend of Track C (**D**). The blue and red circles show the hind-leg and fore-leg strikes, respectively. The dashed red lines show the average of peaks of signals due to fore-leg strikes. The blue dashed lines show the average of peaks of signals due to hind-leg strikes.

Figure 5 illustrates the anterior-posterior accelerations on Tracks A and C (a sample of one greyhound).

The anterior-posterior accelerations versus time of greyhounds galloping on Track A are plotted in Figure 5A,B. The negative peaks in anterior-posterior acceleration are associated with the fore-leg strikes and the positive peaks are due to hind-legs strikes, as mentioned earlier. Comparing the signals resulting from the fore-leg strikes on the bend with those of the Home straight showed a significant difference [t(4) = −8.47 *p* = 0.002].

Additionally, comparing the magnitude of anterior-posterior acceleration signals due to fore-leg strikes with those of hind-leg strikes, on both the Home straight and the bend, showed a significant difference [t(4) = 3.31 *p* = 0.02 & t(4) = 3.2 *p* = 0.02]. Higher levels of G-forces due to hind-leg strikes than the fore-leg one are consistent with the primary role of the hind-leg in powering locomotion, evidenced in the literature [23,39,40]. For instance, Hickman argued that when greyhounds are galloping, the fore-legs are acting like a pivot, while the hind-legs provide the propulsive force [23].

Comparing the anterior-posterior acceleration of hind-leg strikes on the bend with the Home straight did not show a significant difference [t(4) = 0.96 *p* = 0.2].

The anterior-posterior accelerations versus time of greyhounds galloping on Track C are plotted in Figure 5C,D. Comparison of the G-forces from the fore-leg strikes on the bend with those on the Home straight showed a significant difference [t(4) = −22.7 *p* = 0.00009].

Comparison of the magnitude of anterior- posterior acceleration due to fore-leg strikes on the Home straight with those of hind-leg strikes showed a significant difference [t(4) = 14.3 *p* = 0.0003]. However, when the IMU signals of hind-leg strikes on the bend were compared with those of fore-leg, no significant difference was observed [t(4) = 1.17 *p* = 0.16].

Comparison of the the anterior-posterior acceleration of hind-leg strikes on the bend with the Home straight, showed a significant difference [t(4) = 2.31 *p* = 0.05].

In order to see whether surface compliance had any effect on the dynamics of greyhound galloping, the anterior-posterior and dorsal-ventral accelerations for both Track A and C were compared. Furthermore, the signals of galloping on the Home straight were compared to eliminate other factors, which might affect the results, for example the turning effect.

Anterior posterior accelerations due to fore-leg strikes on Track A were slightly higher than those on Track C but were not significantly different [t(4) = 1.45 *p* = 0.12]. Likewise, when the dorsal-ventral accelerations were compared, the values were not statistically different [t(4) = 2.24 *p* = 0.06]. Similarly, no significant difference between the anterior-posterior acceleration due to hind-leg strikes was seen between the two tracks [t(4) = 0.93 *p* = 0.2].

The results of Experiment 2 are tabulated in Table 2:

### 4.3. FFT Analysis

FFT is used to study the temporal characteristics of gaits that is, the stride frequency and the step frequency. The first and second harmonics of dorsal-ventral or anterior-posterior acceleration are due to the stride and step frequency, respectively [14].

**Figure 6:**
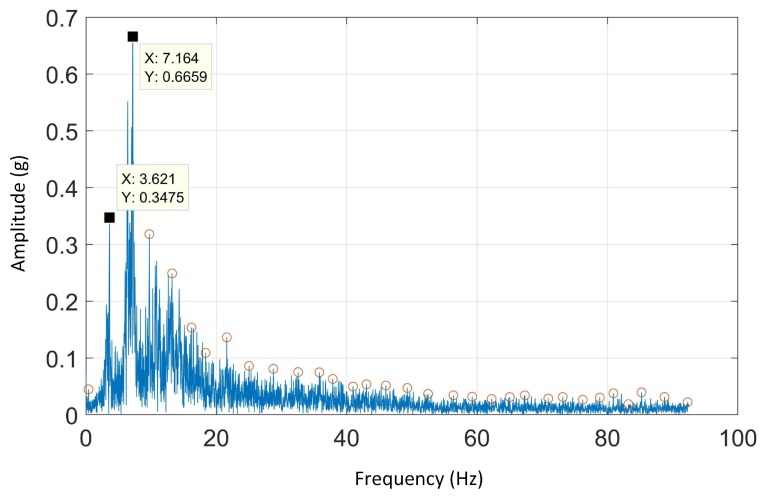
Fast Fourier transform (FFT) spectral analysis of anterior-posterior acceleration of Track A.

Figure 6 depicts the FFT spectral analysis on anterior-posterior acceleration for a sample of one dog on Track A.

The average of stride and step frequencies in all tracks was around 3.5 Hz. These results were consistent with the measured stride frequencies obtained using HFR videos and literature [41]. The stride frequencies of Track A, B, C and D are tabulated in Table 3 below:

### 4.4. Morlet Wavelet Spectral Analysis

Wavelet spectral analysis is another strong signal-processing tool with various applications in different fields. The main advantages of wavelet analysis over FFT analysis is that wavelets are localised in both time and frequency whereas the FFT is localised only in frequency. In this section, the results of wavelet analysis on dorsal-ventral and anterior-posterior accelerations for all race tracks are given and compared with the time-domain signals.

#### 4.4.1. Track A

**Figure 7:**
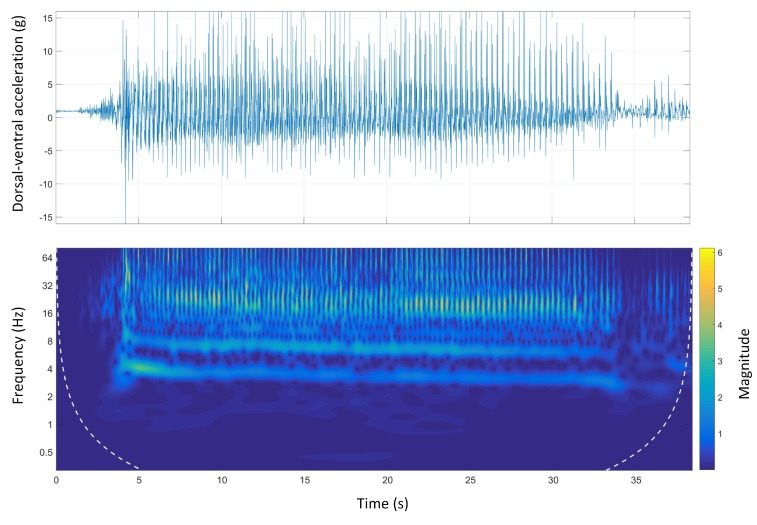
Dorsal-ventral accelerations and Morlet wavelet power spectrum at Track A.

Figure 7 shows the time-domain and wavelet transform plots for the dorsal-ventral accelerations on Track A.

The stride and step frequencies of the whole run in the spectrum which are produced by two main temporal periodicities are visible. The stride and the step frequency of the acceleration phase, (the first four seconds of the run), was 4 Hz and 8 Hz, respectively. During the acceleration phase, the stride frequency and magnitude were higher than the rest of the course. It is clear that the magnitude of the signals was decreasing during the course. The deceleration phase was also clear as the step and stride frequency as well as the magnitude of the signals sharply decreased at the end of the course.

**Figure 8:**
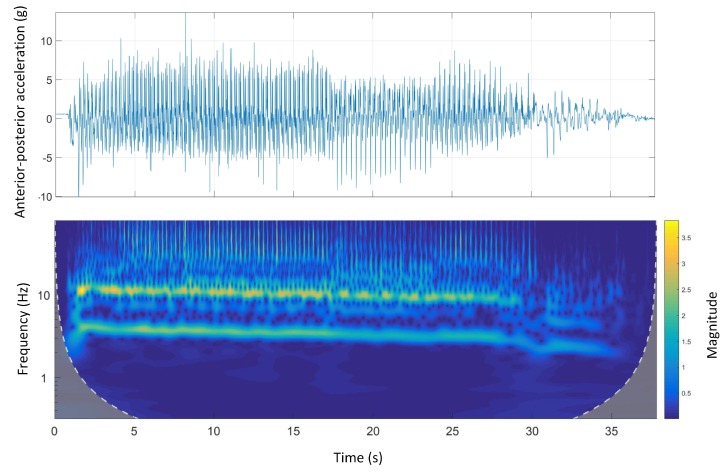
Anterior-posterior accelerations and Morlet wavelet power spectrum at Track A.

Figure 8 shows the time-domain and wavelet transform plots for the anterior-posterior accelerations of Track A.

A similar pattern to dorsal-ventral accelerations was seen in anterior-posterior accelerations. There was a clear high-frequency high-magnitude signal (10 Hz to 12 Hz) in the wavelet power spectrum. Additionally, comparing the wavelet spectral analysis of Track A with other tracks, the energy expenditure (magnitude) was irregular. This might be due to a inconsistent galloping environment that is, track design mainly the track surface as Track A is the only track with a natural grass surface.

#### 4.4.2. Track B

**Figure 9:**
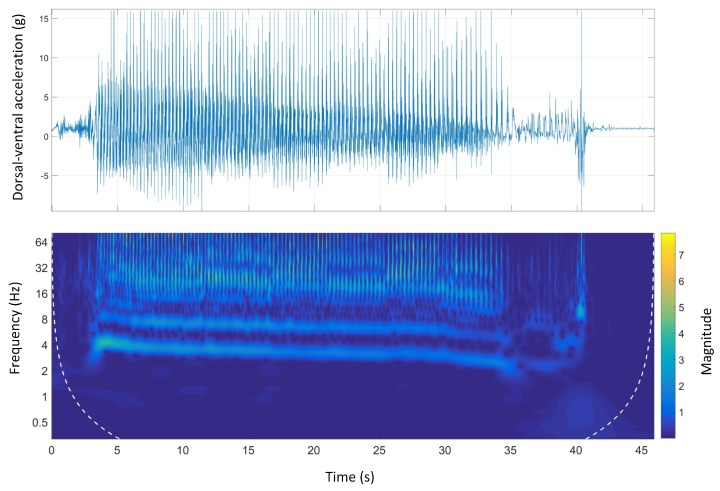
Dorsal-ventral accelerations and Morlet wavelet power spectrum at Track B.

Figure 9 shows the time-domain and Morlet wavelet power spectrum plots for the dorsal-ventral accelerations of Track B. The stride and the step frequency of the acceleration phase was 4 Hz and 8 Hz, respectively. Similar to Track A, the stride and step frequency decreased during the course. The sharp spike in the time-domain signal was due to catching the mechanical lure in the catching pen which is also clear in the wavelet plot.

**Figure 10:**
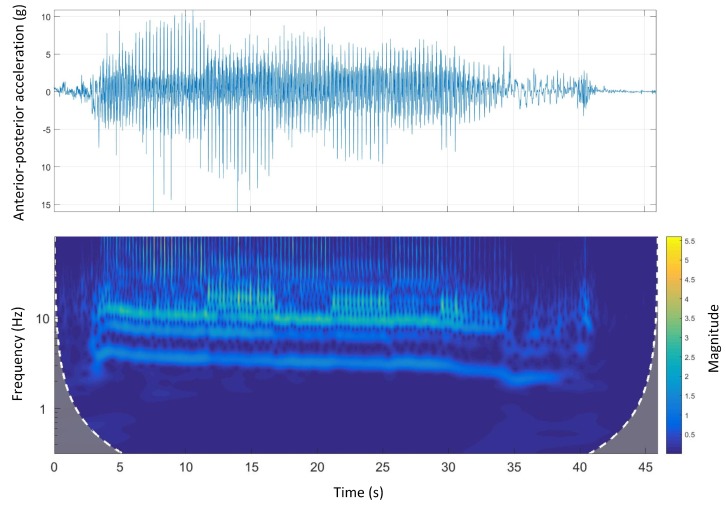
Anterior-posterior accelerations and Morlet wavelet power spectrum at Track B.

Figure 10 shows the time-domain and wavelet transform plots for the anterior-posterior accelerations of Track B. The wavelet power spectrum of the anterior-posterior acceleration shows that the fluctuation of signal magnitude was consistent. This might be because this track is semi-circular with no abrupt changes in transition from straight to bend sections. In addition, at the end of the course, the animal was walking before a complete stop. The difference in the gait characteristics, mainly the stride frequency, is clear in the wavelet power spectrum between the walking and galloping gait.

#### 4.4.3. Track C

**Figure 11:**
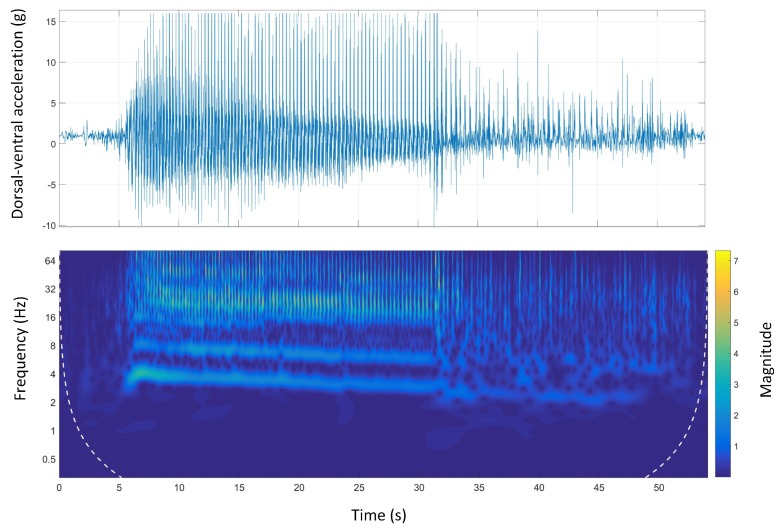
Dorsal-ventral accelerations and Morlet wavelet power spectrum at Track C.

Figure 11 shows the time-domain and Morlet wavelet power spectrum plots for the dorsal-ventral accelerations of Track C. Similar to Track A and B, the magnitude and frequency of the signals were decreasing. Also, the animal walking in the catching pen area before stopping was clear in the wavelet spectrum as the frequency and power of the signals decreased.

**Figure 12:**
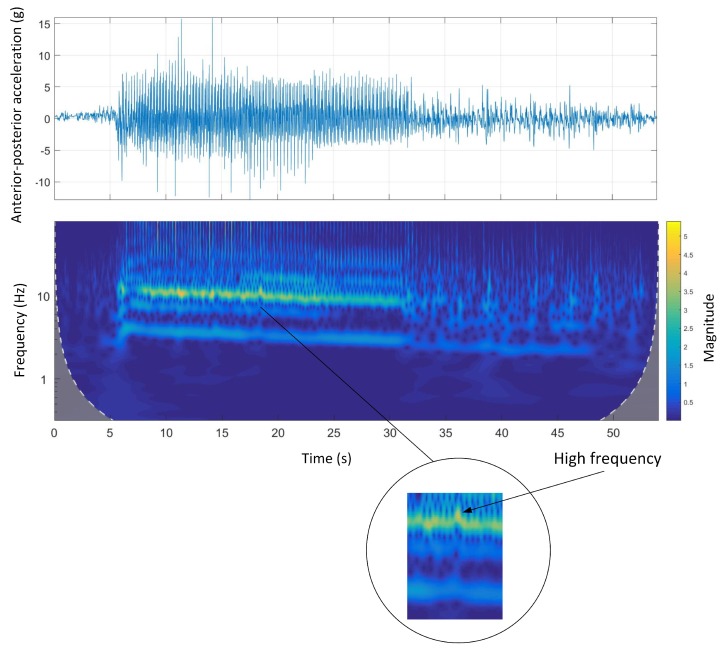
Anterior-posterior accelerations and Morlet wavelet power spectrum at Track C.

Figure 12 shows the time-domain and Morlet wavelet power spectrum plots for the anterior-posterior accelerations of Track C. As can be seen, there was a sharp increase in both frequency and magnitude when the animal exited the bend section and entered the following straight section.

#### 4.4.4. Track D

**Figure 13:**
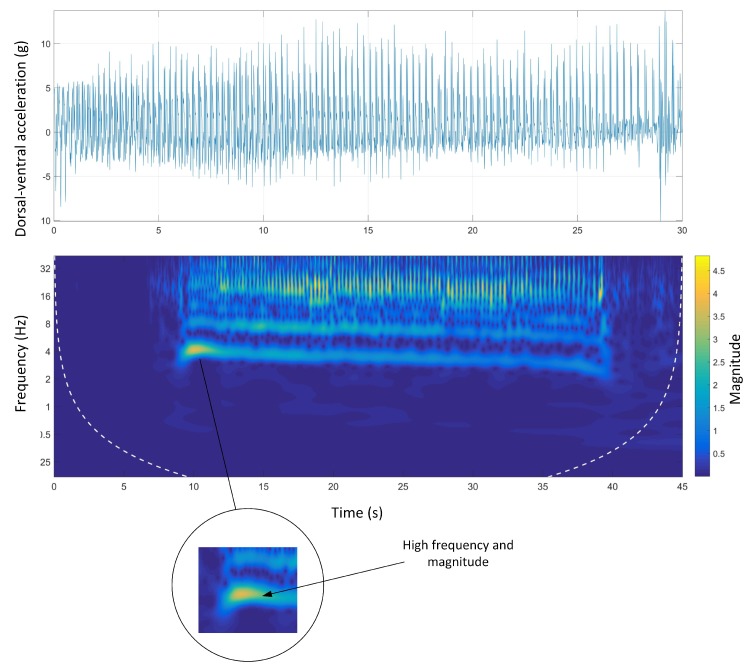
Dorsal-ventral accelerations and Morlet wavelet power spectrum at Track D.

Figure 13 shows the time-domain and Morlet wavelet power spectrum plots for the dorsal-ventral accelerations of Track D. Figure 13 shows that the stride frequency and stride magnitude of the acceleration phase of the run was high and then significantly decreased during the rest of the race.

**Figure 14:**
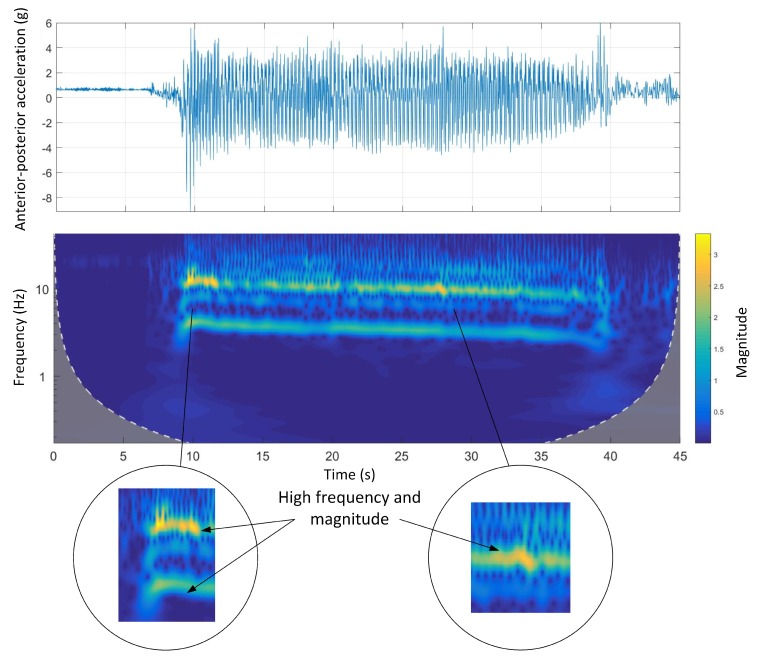
Anterior-posterior accelerations and Morlet wavelet power spectrum at Track D.

Figure 14 shows the time-domain and Morlet wavelet power spectrum plots for the anterior-posterior accelerations of Track D. In Track D, the greyhound had a high energy acceleration which is clearly seen in both the time-domain plot and the wavelet spectrum. The abrupt drop of acceleration magnitude while entering from the straight section to the bend was also clear.

## 5. Discussion

In this paper, the galloping gait characteristics of agile canines (racing greyhounds) were analysed using accelerometry. In the results obtained from Experiment 1, it was found that the negative and positive peaks in the anterior-posterior accelerations were due to fore-leg and hind-leg strike. The positive peaks of the dorsal-ventral accelerations were also due to hind-leg strike. It was also observed that the hind-leg accelerations in the dorsal-ventral direction were almost fifteen times the animals weight. This fact is consistent with the high rate of hind-leg injuries evident in the literature [23,27,28,38], as well as its main role in powering the locomotion [36].

The objectives set for Experiment 2 were to examine whether different sections of the race track (i.e., bends and straight sections) and the surface type affect the galloping dynamics of greyhounds. To study the turning dynamics, the anterior-posterior and dorsal-ventral acceleration of the steady-state of greyhounds galloping on the Home straight were compared with those of the bend. Track A and C were selected for this experiment. The peaks of dorsal-ventral accelerations acting on hind-legs on bends and straight sections in Track A and C were equal to 15 G. In other words, a load fifteen times greyhound body weight were acting on the animal each time the hind-legs came into contact with the ground. This correlates well with high rate of hind-leg injuries [26,27,38]. The peaks of dorsal-ventral accelerations acting on fore-legs on bends, were significantly higher than those of Home straight (T-test on the signals on Track A [t(4) = 8.14 *p* = 0.002] and on Track C [t(4) = 5.7 *p* = 0.005]). It was also mentioned in the literature that turning is potentially more hazardous than straight galloping [27,38]. The results obtained of this research support this. Higher repetitive impulsive G-forces were acting on fore-legs while animals were turning than were galloping on the Home straight.

The second objective of Experiment 2 was studying the possible effect of surface compliance on greyhounds galloping dynamics. To do so, the anterior-posterior and dorsal-ventral accelerations of greyhounds galloping on the Home straight of Track A were compared with those of Track C. Anterior-posterior accelerations due to fore-leg strikes on Track C were slightly higher than those of Track A but were not significantly different [t(4) = 1.45 *p* = 0.12]. Likewise, the dorsal-ventral accelerations were compared and the values were not statistically different [t(4) = 2.24 *p* = 0.06]. Similarly, no significant difference between the anterior-posterior acceleration due to hind-leg strikes was seen between the two tracks [t(4) = 0.93 *p* = 0.2]. These results were despite the fact that the stiffness coefficient of grass is almost three times lower than that of the sand obtained using a standard drop test in a previous study [9,42]. This suggests that either surface compliance does not impact greyhound galloping dynamics or the accelerometry with only one single IMU cannot quantitatively compare the difference between surface types. The former seems more promising based on the literature that the surface types correlate with the rate and severity of injuries. For instance, Prole mentioned that on the grass surface 95% of all the reported foot injuries were on toes, while only 6% of them were on tarsal bones. This is despite the fact that tarsal injuries are frequently seen on sand tracks [27,43]. Moreover, in a previous study conducted by Hayati et.al [28] where the greyhound’s hind-leg and the underneath surface were mathematically modelled, it has proven that surface compliance affects the dynamics of the hind-leg during galloping gait.

The stride frequency and stride length are two important speed indicators in legged locomotion [31]. Applying FFT on dorsal-ventral accelerations would obtain the dominant harmonics of the signals which represents the stride frequency (the first harmonic). The results of the FFT analysis on the dorsal-ventral acceleration on Track A, B, C and D showed two dominant harmonics. As mentioned, the first one is associated with the stride frequency and the second one might be the step frequency which is also seen in equine gait characteristics [14].

The results obtained by applying FFT on the aforementioned tracks are tabulated in Table 3. As it can be seen, the stride frequencies were similar (3.5 Hz).Assuming the stride length is available, an approximate speed of animals can be calculated. This approach can be used to re-visit the fact that greyhounds do not slow down while turning [40]. Based on our preliminary data, where we measured the stride-length and stride frequency on Track E, greyhounds slow down on bends as the stride-length were shorter on bends than on straight section, while the stride frequencies were similar [8]. However, more data are required to support this observation.

Although some temporal galloping gait characteristics were revealed using FFT analysis, more information was still required to have a better understanding of the galloping gait. Accordingly, Morlet wavelet spectral analysis was applied on both anterior-posterior and dorsal-ventral accelerations for all tracks.

The stride and step frequencies were clear in the wavelet spectrum of the anterior-posterior accelerations on all tracks. The acceleration and deceleration phases were also clear on all tracks. The acceleration phase (three to four seconds of the beginning of the race) is associated with high frequency/high magnitude stride frequency, which was clearly seen in Track D (Figure 13). This was in contrast with the deceleration phase, which was associated with low frequency/low magnitude stride frequency, happening at the end of the course. The stride and step frequencies of animals were decreasing on all the tracks suggesting animal speed reduction due to fatigue during the course.

Track A showed a very inconsistent stride magnitude during the run compared to other tracks (Figure 7). This may be because of an inconsistent track environment as Track A was the only track with a grass surface. By contrast, Track B, had a more consistent stride magnitude suggesting a smoother run. This might be due to the design of the track which is a semi-circular track with no sudden transition from straight to bend and the other way around.

There were two clear abrupt changes observed in the anterior-posterior accelerations on Track C and D when the animal entered the bend section shown in Figure 12 and Figure 14, respectively. The abrupt changes suggest that the animal either changed the running path to a larger radius to avoid the centrifugal force and jerk on bends [11] or tried to maintain its vision of the mechanical lure while turning. The latter behaviour is one of the reasons for greyhounds clustering while approaching bends [44] and the consequent accidents on bends [27].

It should also be acknowledged that due to limitations such as available greyhounds and industry restrictions, not all variables were controlled or recorded in this study. Other variables could have affected the results such as age, sex, weight, training and race experience which ideally should be recorded and considered in analysis could have affected the results. A statistical analysis conducted in a recent publication in 2019 [45] showed that age is significantly different between injury types. No evidence was reported that correlate speed and injury, however, these variables can be considered and will be investigated in future work where possible. The research was also restricted by the animal ethics approval not to run more than one greyhound in each trial. Accordingly, all the aforementioned factors may have affected the results obtained, reported and discussed in this work.

## 6. Conclusions

The galloping gait characteristics of agile canines (racing greyhounds) were analysed in this paper. Commercial and in-house IMU packages were used and FFT and Morlet wavelet spectral analysis were applied to the anterior-posterior and dorsal-ventral axis accelerations. It was observed that the information obtained by analysing the time-domain signals was relatively limited. This led to applying FFT and wavelet spectral analysis to acceleration data. The FFT results showed two dominant harmonics evident in all the data associated with the stride and step frequencies. The wavelet spectral analysis also showed that stride frequency reduces throughout the race, which might suggest speed reduction. In addition, the wavelet spectrum of some tracks showed an abrupt change in signal when animals entered the bend. It can be concluded that utilising an IMU sensor as well as the methodologies conducted in this work is beneficial in understanding the unexplored and complex gait of agile canines. The result can further contribute to racing animals safety and welfare by detecting the hazardous locations in their racing environment. With more data on future research, more statistical analysis can be carried out and therefore a more rigorous conclusion can be derived. 

## Figures and Tables

**Table 1:** Track specifications based on available survey data.

Track	Bend Radius (m)	Straight Length (m)	Cross-Fall Bend (%)	Cross-Fall Straight (%)	Surface	Number of Dogs
Track A	81	n/a	0	0	Grass	4
Track B	63	n/a	4.81	n/a	Sand	4
Track C	51	113	7.40	3.50	Sand	4
Track D	51	44	8.32	3.84	Sand	18
Track E	50	53	6.23	4.42	Sand	1

**Table 2:** Average of peaks for anterior-posterior and dorsal-ventral accelerations on Tracks A and C.

Location on the Track	Track A (Sand)	Track C (Grass)
Anterior-posterior Acc|Fore-leg|Bend	−5.0 ±−0.8 G	−5.4 ±−0.2 G
Anterior-posterior Acc|Fore-leg|Straight	−8.0 ±−0.7 G	−8.7 ±−0.3 G
Anterior-posterior Acc|Hind-leg|Bend	6.6 ± 1.3 G	6.0 ± 0.7 G
Anterior-posterior Acc|Hind-leg|Straight	5.4 ± 1.3 G	4.7 ± 0.6 G
Dorsal-ventral Acc|Fore-leg|Bend	6.2 ± 0.79 G	6.4 ± 0.74 G
Dorsal-ventral Acc|Fore-leg|Straight	4.4 ± 0.26 G	4.7 ± 0.42 G
Dorsal-ventral Acc|Hind-leg|Bend	15.0 G	15.0 G
Dorsal-ventral Acc|Hind-leg|Straight	15.0 G	15.0 G

**Table 3:** The average of stride frequencies of greyhounds galloping on Track A, B, C and D.

Track	Stride Frequency (Hz)
A	3.5 ± 0.11
B	3.4 ± 0.14
C	3.4 ± 0.15
D	3.3 ± 0.30

## Abbreviations

The following abbreviations are used in this manuscript:

CFL	compressed flight phase
CoM	center of mass
CWT	continuous wavelet transform
DFT	discrete Fourier transforms
EFL	extended flight phase
FFT	fast Fourier transform
GPS	global positioning system
GRF	ground reaction force
HFR	high frame rate
iKMS	integrated kinematic measurement system
IMU	inertia measurement unit
LF	left fore-leg
LH	left hind-leg
RF	right fore-leg
RH	right hind-leg
